# Medical Miscellany

**Published:** 1854-06

**Authors:** 


MEDICAL MISCELLANY.—From recent investigations among the tombs of the ancient Egyptians, it has been discovered that they were acquainted with the use of nitrate of silver as an indelible ink.——— Dr. Snooks never trifled with disease. His directions were—bleed the north ward and blister the south ward to day; and blister the north and bleed the south to-morrow.———It has been found by actual experiment, performed near London, that in animals a greater degree of fattening was obtained from a less amount of food where cod liver oil was used. Hogs took two ounces, sheep one, and cattle from a quarter to three-quarters of a pint per day, and paid better than any in market.———There was 53 suicides in New York city last year.———A lumberman in Maine recently found a stone weighing 33 pounds in the centre of a smooth and handsome tree, which he felled.———A sailor on a ship in Boston harbor, recently fell to the deck from a height of eighty feet, without injuring himself.——— Dr. Spear, of Florida, raised 20,000 lemons from 250 trees. He is the largest lemon grower in the country.———The last U. S. census shows that there were then four hundred and thirty American women above one hundred years of age.———Forty-three deaths by small pox in New York city in one week. This disease is upon the increase throughout the country. Prepare for it, and remember that only one vaccination is not always a sure protection.———Fourteen admissions into the N. Y. State Lunatic Asylum last year from effects of spiritual rappings.———It is stated that there are ten or twelve cases of cancer in the London Hospitals, all the result of excessive smoking.———A paper says that hydrophobia may be prevented by washing the recent wound with a solution of two tablespoonfulls of powdered chloride of lime, to half pint of water.———The city authorities of Boston kill all the dogs found in the streets, generally with strychnine; some cases of hydrophobia had occurred.

